# Standardization of the Cirrhosis Admission Process and Its Impact on Inpatient Management and Patient Outcomes

**DOI:** 10.1111/liv.70674

**Published:** 2026-05-05

**Authors:** Laura E. Lavette, Hannah Laird, Ashley Chipoletti, Mira Sridharan, Jessica J. Dreicer, Andrew Barros, Zachary Henry

**Affiliations:** ^1^ Department of Medicine University of Virginia School of Medicine Charlottesville Virginia USA; ^2^ Department of Medicine Boston University School of Medicine Boston Massachusetts USA; ^3^ Division of Pulmonary and Critical Care University of Virginia School of Medicine Charlottesville Virginia USA; ^4^ Division of Gastroenterology and Hepatology University of Virginia School of Medicine Charlottesville Virginia USA

**Keywords:** cirrhosis, order set, quality improvement

## Abstract

**Introduction:**

An increase in cirrhosis‐related hospitalizations has highlighted opportunities for improved patient care and standardization of practice. We created a cirrhosis admission order set with the goal of promoting guideline‐directed care and improving patient outcomes.

**Methods:**

We developed and implemented a cirrhosis order set that included dietary recommendations, diagnostic paracentesis orders and studies, common complications of portal hypertension and educational materials at time of discharge. We used a retrospective cohort design to compare post‐intervention process measures and outcomes.

**Results:**

The use of the order set resulted in a significant increase in low sodium diet (93% vs. 37%, *p* = 0.001), high protein diet (91% vs. 37%, *p* = 0.001) and diagnostic paracentesis (46% vs. 26%, *p* = 0.001) performed during admission. There was also a significant reduction in time to diagnostic paracentesis (460 min vs. 1210 min, *p* = 0.03) and less all‐cause Emergency Department visits (32% vs. 46%, *p* = 0.006) in the order set group. There was no significant impact on length of stay, readmission rates or in‐hospital mortality.

**Discussion:**

The use of a standardized order set improves inpatient management of cirrhosis and encourages guideline‐directed care. This order set could easily be replicated at other institutions or among non‐cirrhotic patient populations. Future work should focus on further standardization of the discharge process with the goal of having a more tangible impact on length of stay and readmission rates.

## Lay Summary

Cirrhosis is associated with increased hospitalizations and healthcare costs. Use of a cirrhosis admission order set improves evidence‐based inpatient management of cirrhosis and is associated with decreased all‐cause Emergency Department visits. Future work should focus on further standardization of the cirrhosis discharge process.

**Abbreviations**
AASLD: American Association for the Study of Liver Diseases
AKI: Acute Kidney Injury
ALD: Alcohol‐related Liver Disease
BMI: Body Mass Index
CI: Confidence Interval
ED: Emergency Department
EMR: Electronic Medical Record
GIB: Gastrointestinal Bleed
HE: Hepatic Encephalopathy
HR: Hazard Ratio
ICU: Intensive Care Unit
IQR: Interquartile Range
IRB: Institutional Review Board
LOS: Length of Stay
MASH: Metabolic Dysfunction‐Associated Steatohepatitis
MELD: Model for End‐Stage Liver Disease
OR: Odds Ratio
QI: Quality Improvement
RR: Relative Risk
SBP: Spontaneous Bacterial Peritonitis
UVA: University of Virginia

## 1 Introduction

Liver disease accounts for over two million deaths annually and is currently the eleventh leading cause of death globally [1]. The prevalence of cirrhosis has been increasing since at least 2012, in part due to an uptake in alcohol consumption and metabolic syndrome related diseases such as obesity and diabetes [2]. Unsurprisingly, there has been an associated increase in cirrhosis‐related hospitalizations and healthcare costs [3, 4]. Readmission rates are also high, with prior studies showing that up to 25% of patients with decompensated cirrhosis will be re‐admitted within 30 days [5, 6].

The increasing prevalence of liver disease has exposed certain shortcomings in the healthcare system. Previous data has indicated that around 50% of patients with ascites undergo paracentesis during admission, while only 30% are provided a low sodium diet [7, 8, 9]. A retrospective study in Virginia looked at cirrhosis patients hospitalized with hepatic encephalopathy (HE); only 22% received a complete diagnostic work‐up and 77% did not undergo paracentesis to rule out spontaneous bacterial peritonitis (SBP). In contrast, serum ammonia level (which is not supported by current HE guidelines) was monitored in 95% of patients [10]. A multicenter audit found that almost 70% of patients with new HE were not discharged with a prescription for lactulose [11, 12]. This is a short yet comprehensive list of quality care gaps that likely lead to compromised patient care.

Previous efforts aimed at improving patient care in cirrhosis have included early intervention, interdisciplinary teams and focus on quality improvement (QI). In 2019, The Practice Metrics Committee of the American Association for the Study of Liver Diseases (AASLD) established evidence‐based quality measures for adult patients with cirrhosis to promote high‐value care [7]. With these quality measures in mind, we developed a cirrhosis order set with the goal of standardizing the admission process, encouraging guideline‐directed medical care and improving inpatient management of cirrhosis.

## 2 Methods

### 2.1 Order Set Design

Our study is based on the results of a QI project undertaken to improve adherence to guideline recommended therapies in cirrhosis. First, we retrospectively assessed a group of patients admitted to the University of Virginia (UVA) Medical Center with a diagnosis of cirrhosis from January 2022 to August 2023. Using this data, we were able to identify pertinent outcomes and co‐morbid variables of interest prior to our intervention.

**FIGURE 1:**
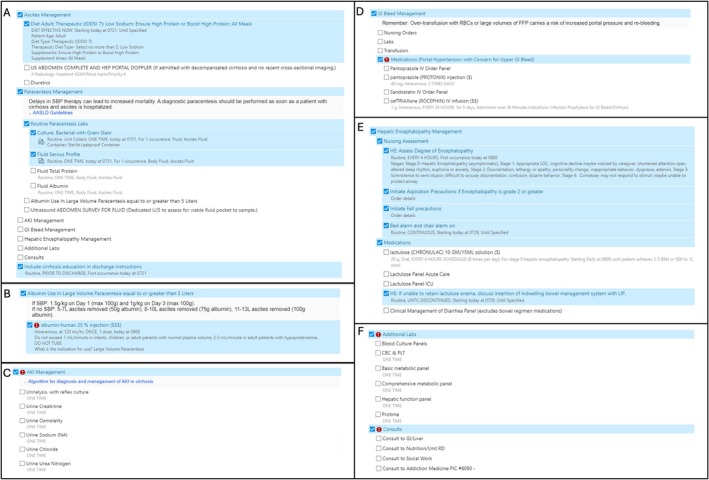
Cirrhosis order set categories including (A) Pre‐selected categories, (B) Albumin for Large Volume Paracentesis (LVP), (C) Acute Kidney Injury (AKI) Management, (D) Gastrointestinal Bleed (GIB) Management, (E) Hepatic Encephalopathy Management, (F) Labs and Consults.

Our order set, titled ‘Cirrhosis Management Panel,’ was created as a stand‐alone order set and embedded into the general admission order set. It consists of eight categories related to common complications of portal hypertension. Pre‐selected categories include diet orders, paracentesis labs and cirrhosis education at time of discharge. Additional categories include ascites, acute kidney injury (AKI), gastrointestinal bleed (GIB), HE and consults (Figure 1). The order set includes embedded links to AASLD guidelines as well as proposed algorithms for more complex clinical decisions (i.e., how to approach AKI in cirrhosis). The order set was reviewed and edited by a multidisciplinary team including Internal Medicine Residents, Hospitalists, Health Informatics Specialists and Hepatologists. The order set went live in January 2024. Over the next 12 months, residents and hospitalists were educated on the order set through business meetings and email reminders.

### 2.2 Study Design

Analysis of our quality intervention was designed as a cohort study with pre‐defined outcomes and exposures. Patients were captured for analysis at time of admission and followed out to 30 days post‐discharge. All patients were admitted to UVA Medical Center, a tertiary care referral hospital in central Virginia. We collected information on patients with a diagnosis of cirrhosis admitted to the general medicine service between February 1, 2024 and January 10, 2025. Despite having pre‐intervention data available, we intentionally compared cirrhosis patients with and without order set use over the same time period to eliminate confounding variables such as provider turnover, patient volume and/or changes in hospital policy.

**FIGURE 2:**
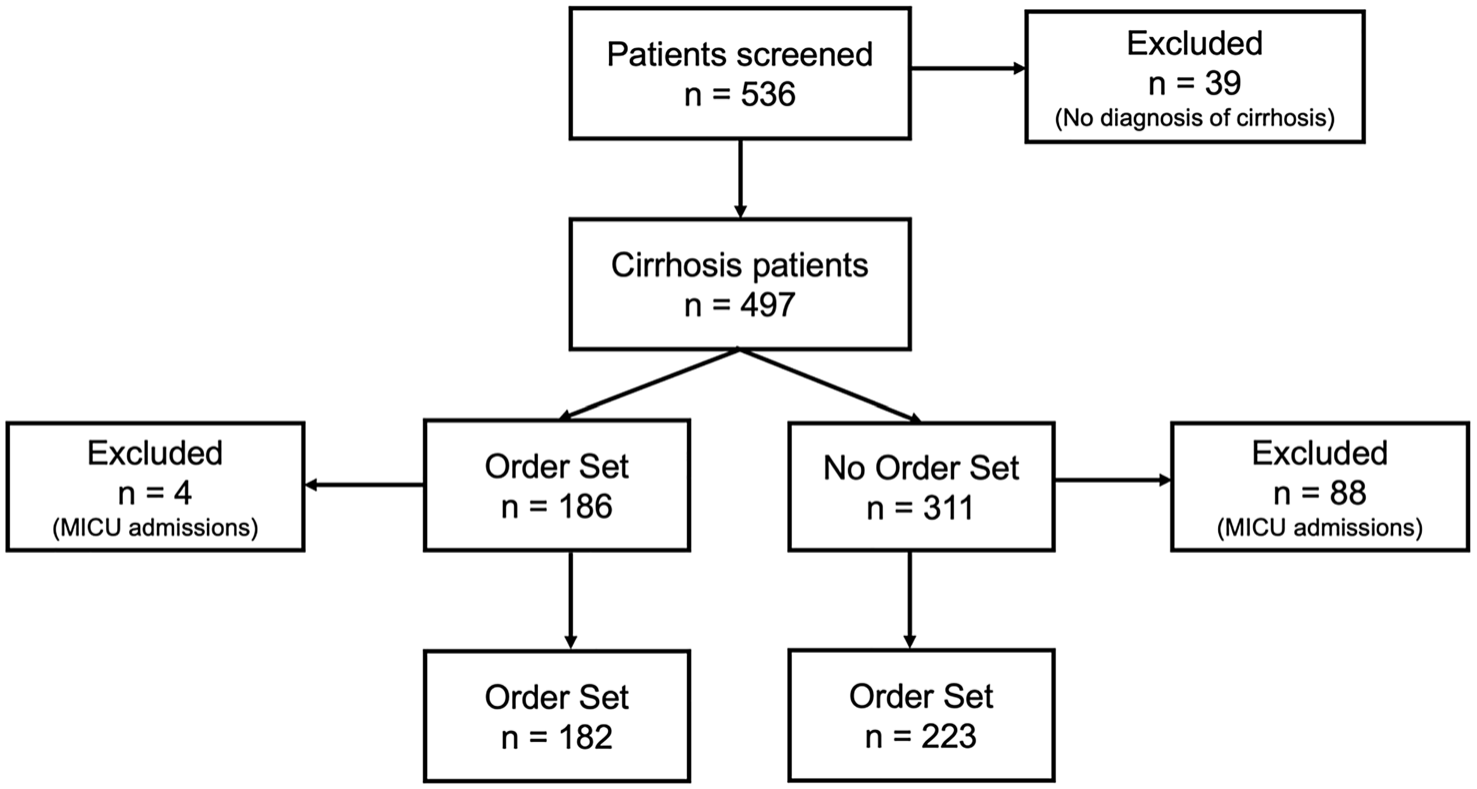
Study flow diagram including patients initially screened and those excluded from final analysis.

Inclusion criteria comprised all patients with a diagnosis of cirrhosis at time of admission who were over the age of 18. Multiple admissions by the same patient were included as separate patient encounters to account for variability in care (i.e., order set use, timing of paracentesis, etc.). Exclusion criteria included misdiagnosis of cirrhosis on chart review or admission to a non‐general medicine service, including those initially admitted to the Intensive Care Unit (ICU) (Figure 2). The diagnosis of cirrhosis was based on clinical criteria including imaging, lab testing, history of chronic liver disease and evidence of portal hypertension or liver biopsy when available. We included patients with compensated cirrhosis because a low sodium, high protein diet is still relevant in this population [13].

### 2.3 Exposure Assessment

Our exposure of interest in this study was defined as utilization of the order set. We were able to identify which patients had the order set used through LogicStream software. Based on our prior retrospective analysis, we also determined co‐morbid variables for capture at time of admission: baseline demographics, presence of decompensated cirrhosis, complications of portal hypertension, co‐morbid medical diagnoses and liver function blood tests.

### 2.4 Outcome Measures

The aim of the order set was to standardize and improve inpatient management of cirrhosis. Diagnostic paracentesis within 24 h of admission is associated with reduced mortality and hospital length of stay (LOS) [14]. Low sodium and high protein diets have also been linked to decreased mortality in the cirrhosis population [15, 16]. Moreover, timely paracentesis and nutrition are outlined in the current AASLD guidelines [13, 17]. Therefore, our primary process outcomes included utilization of a low sodium and high protein diet, performance of a diagnostic paracentesis during admission and time to diagnostic paracentesis.

Performance of diagnostic paracentesis was defined as successful collection of ascites fluid sent for cell count +/− culture data. Time to diagnostic paracentesis was defined as time (in minutes) lapsed between time of admission (i.e., admission order placed) and time of procedure (i.e., labs sent off for analysis). Time from admission (as opposed to time from ED arrival, etc.) was selected based on quality metrics derived from the AASLD and clinical practice guidance discussions [9, 18]. For the order set group, we included analysis from time of admission for all patients and then repeated analysis only using patients who had a paracentesis performed after the order set was entered. Our primary clinical outcomes included in‐hospital mortality and LOS.

Our order set also contained cirrhosis discharge instructions (education on common complications of cirrhosis, important medications and return precautions), so secondary clinical outcomes included visits to the Emergency Department (ED), readmissions and death within 30 days of discharge. We also performed sub‐analyses looking at liver‐specific causes related to these outcomes. Any ED visit or hospitalization at our institution was captured, but visits to an outside hospital ED were not possible to identify using the Electronic Medical Record (EMR); this was similar for readmissions and patient deaths. All data were captured using the UVA Institutional Data Warehouse. Missing data for co‐variables were left blank and excluded from analysis.

### 2.5 Statistical Analysis

Demographics are represented using median with interquartile range (IQR) for continuous variables and percentages for categorical variables. Based on our defined primary exposure, differences between groups were assessed using Wilcoxon rank sum analysis for continuous variables and chi‐square or fisher's exact test for categorical variables. Relative risk (RR) based on the primary exposure was calculated for statistically significant outcomes of interest. To determine independent predictors of outcomes, univariable logistic regression was used to assess exposure groups and co‐morbid variables. Any variable with a *p*‐value < 0.1 was included in a multivariable logistic regression analysis using backward selection. Time‐to‐event analysis using cox proportional hazards (CPH) model was employed to assess independent predictors impacting time to diagnostic paracentesis. For the primary outcomes related to performance of paracentesis, only patients with documented ascites were included in the regression models. A *p*‐value threshold < 0.05 was considered statistically significant. SAS version 9.4 was used for statistical analysis. Ethical approval for this QI project was approved by the UVA Institutional Review Board (IRB) as an expedited review, and the need for individualized consent was waived because all data were anonymized. SQUIRE 2.0 and STROBE checklists were used in the development of the manuscript [19, 20].

## 3 Results

### 3.1 Baseline Characteristics

A total of 405 patients with cirrhosis were admitted to the general medicine service during the period of data collection. Over half (56%) of the patients were admitted for complications of liver disease, which was a likely driver of order set use. Patients had varying etiologies of liver disease, with the majority (60%) being alcohol‐related. Median LOS was 5 days, and 10% of patients were transferred to the ICU at some point during their hospitalization. Nearly half (45%) of the patients had the order set used during their admission (Table 1). We were unable to break down order set use by provider type (resident physician, hospitalist, etc.), which may have influenced how and when the order set was used.

**TABLE 1:** Demographic data of entire cohort.

Variables	Overall cohort (*n* = 405)
Age (years)	58 (49–66)
MELD‐3.0	19 (10–26)
INR	1.5 (1.3–2.0)
Bilirubin	2.4 (1.2–6.0)
Creatinine	1.0 (0.7–1.4)
Sodium	135 (131–138)
Albumin	3.0 (2.6–3.4)
Gender *n* (% male)	250 (61)
BMI	30 (26–35)
Admit for liver disease complication *n* (%)	225 (56)
Admit for EV bleed *n* (%)	22 (5)
Admit for ascites *n* (%)	62 (15)
Admit for HE *n* (%)	76 (19)
Admit for AKI *n* (%)	45 (11)
Aetiology of liver disease	
ALD *n* (%)	242 (60)
MASH *n* (%)	92 (23)
AIH *n* (%)	8 (2)
Viral hepatitis *n* (%)	19 (5)
Cryptogenic *n* (%)	24 (6)
Other *n* (%)	20 (5)
Decompensated *n* (%)	366 (90)
History of ascites *n* (%)	210 (52)
History of SBP *n* (%)	50 (12)
History of HE *n* (%)	240 (59)
ICU transfer *n* (%)	41 (10)
Length of stay (days)	5 (2–8)
Diagnostic paracentesis performed *n* (%)	142 (35)
Low sodium diet order placed *n* (%)	251 (62)
High protein diet order placed *n* (%)	248 (61)
30‐day readmission *n* (%)	134 (35)
30‐day ED encounter *n* (%)	152 (40)
30‐day mortality *n* (%)	14 (4)
In‐hospital mortality *n* (%)	14 (4)
Order set used *n* (%)	182 (45%)
Time to order set (min)	182 (0–397)
Time to paracentesis after order set (min)	118 (−33–968)

### 3.2 Comparison of Groups

There were no significant differences between groups in median age (58 vs. 59, *p* = 0.9), male gender (59% vs. 64%, *p* = 0.3), median body mass index (BMI; 31 vs. 30, *p* = 0.07), ICU transfer (8% vs. 12%, *p* = 0.2) or transfer from an outside hospital (12% vs. 7%, *p* = 0.2). The order set group did have a significantly higher median Model for End‐Stage Liver Disease (MELD) score (20 vs. 18, *p* = 0.008), was more likely to be admitted for a complication of liver disease (66% vs. 47%, *p* = 0.001), more likely to have decompensated cirrhosis (96% vs. 86%, *p* = 0.001) and more likely to have a history of ascites (63% vs. 43%, *p* = 0.001), SBP (16% vs. 9%, *p* = 0.03) or HE (71% vs. 49%, *p* = 0.001). There was also a significant difference in etiology of cirrhosis between groups (*p* = 0.001), with the order set group having more diagnoses of metabolic dysfunction‐associated steatohepatitis (MASH) and cryptogenic cirrhosis and the no order set group having more diagnoses of alcohol‐related liver disease (ALD) and viral hepatitis (Table 2).

**TABLE 2:** Comparison of demographic data between exposure groups.

Variables	Order set (*n* = 182)	No order set (*n* = 223)	*p*
Age (years)	58 (50–66)	59 (48–66)	0.9
MELD‐3.0	**20 (12–28)**	**18 (9–25)**	**0.008**
INR	1.5 (1.3–2.0)	1.5 (1.3–1.9)	0.1
Bilirubin	2.7 (1.4–6.0)	2.3 (1.1–6.3)	0.1
Creatinine	**1.1 (0.7–1.6)**	**0.9 (0.7–1.4)**	**0.01**
Sodium	135 (131–138)	135 (121–137)	0.6
Albumin	**2.9 (2.5–3.2)**	**3.1 (2.7–3.5)**	**< 0.001**
Gender *n* (% male)	107 (59)	143 (64)	0.3
BMI	31 (27–35)	30 (26–35)	0.07
Admit for liver disease complication *n* (%)	**120 (66)**	**105 (47)**	**0.001**
Admit for EV bleed *n* (%)	11 (6)	11 (5)	0.7
Admit for ascites *n* (%)	**40 (22)**	**22 (10)**	**0.001**
Admit for HE *n* (%)	40 (22)	36 (16)	0.2
Admit for AKI *n* (%)	23 (13)	22 (10)	0.4
Aetiology of liver disease			
ALD *n* (%)	92 (51)	150 (67)	**0.001**
MASH *n* (%)	54 (30)	38 (17)	
Viral Hepatitis *n* (%)	4 (2)	15 (7)	
Cryptogenic *n* (%)	16 (9)	4 (4)	
Other *n* (%)	12 (6)	8 (4)	
Decompensated *n* (%)	**175 (96)**	**191 (86)**	**0.001**
History of Ascites *n* (%)	**114 (63)**	**96 (43)**	**0.001**
History of SBP *n* (%)	**30 (16)**	**20 (9)**	**0.03**
History of HE *n* (%)	**130 (71)**	**110 (49)**	**0.001**
Transfer to ICU *n* (%)	14 (8)	27 (12)	0.2
Transfer from Outside Hospital *n* (%)	22 (12)	17 (7)	0.2

*Note:* *p* values < 0.05 were bolded to indicate statistical significance.

### 3.3 Primary Outcomes

It took providers a median of 182 min to order the ‘Cirrhosis Management Panel’ from the time of admission. Patients in the order set group had significantly higher utilization of low sodium (93% vs. 37%, RR 2.51, *p* = 0.001) and high protein (91% vs. 37%, RR 2.46, *p* = 0.001) diets during hospitalization (Table 3). On multivariable analysis, independent predictors of a low sodium diet being ordered included use of the order set (odds ratio (OR) 23.6 [12.1–45.9]) and a history of ascites (OR 6.0 [3.5–10.3]). Independent predictors of a high protein diet included use of the order set (OR 15.2 [8.4–27.4]), a history of ascites (OR 2.2 [1.3–3.7]) and MELD score (OR 1.04 [1.02–1.1]) (Table S1).

**TABLE 3:** Primary and secondary outcomes between exposure groups.

Entire cohort (*n* = 405)				
Variables	Order set (*n* = 182)	No order set (*n* = 223)	*p*	RR
LOS (days)	5 (2–8)	5 (2–8)	0.8	
Diagnostic Paracentesis *n* (%)	**83 (46)**	**59 (26)**	**0.001**	**1.77**
Time to Paracentesis (min)	**460 (161–1401)**	**1210 (245–1826)**	**0.03**	
Low Sodium Diet *n* (%)	**169 (93)**	**82 (37)**	**0.001**	**2.51**
High Protein Diet *n* (%)	**165 (91)**	**83 (37)**	**0.001**	**2.46**
30‐Day Readmission *n* (%)	52 (30)	82 (39)	0.1	
30‐Day ED Visit *n* (%)	**55 (32)**	**97 (46)**	**0.006**	**0.70**
30‐Day Liver Related Readmission *n* (%)	33 (18)	38 (17)	0.8	
30‐Day Liver Related ED Visit *n* (%)	31 (17)	41 (18)	0.7	
30‐Day Mortality *n* (%)	10 (6)	4 (2)	0.06	
In‐Hospital Mortality *n* (%)	4 (2)	10 (4)	0.2	

Only those with ascites (*n* = 210)				
Variables	Order set (*n* = 114)	No order set (*n* = 96)	*p*	RR
Diagnostic Paracentesis *n* (%)^a^	80 (70)	58 (60)	0.15	1.2
Time to Paracentesis (min)^a^	**431 (143–1413)**	**1101 (245–1715)**	**0.03**	
Diagnostic Paracentesis *n* (%)^b^	54 (47)	58 (60)	0.06	
Time to Paracentesis (min)^b^	**641 (357–1750)**	**1101 (245–1715)**	**0.8**	

*Note:* *p* values < 0.05 were bolded to indicate statistical significance.

^a^ Including all patients from time of admission to time of paracentesis.

^b^ Only including patients with paracentesis performed after the Order Set was placed.

The order set group had significantly more diagnostic paracenteses performed during admission (46% vs. 26%, RR 1.77, *p* = 0.001) with shorter median time to paracentesis (460 min vs. 1210 min, *p* = 0.03). After excluding patients without a history of ascites (*n* = 195), we found no significant difference in diagnostic paracentesis between groups (70% vs. 60%, RR 1.2, *p* = 0.15), though there was still an improved time to paracentesis when using the order set (431 min vs. 1101 min, *p* = 0.03). Median time to paracentesis was shorter in the order set group but not statistically significant (641 min vs. 1101 min, *p* = 0.8) when excluding patients who had a paracentesis prior to the order set being used (Table 3). Of the patients with a history of ascites (*n* = 210) who did not undergo paracentesis (*n* = 72, 34%), we found that 68% did not have a large enough pocket deemed safe for paracentesis, 8% had a paracentesis at an outside hospital and it was not felt necessary to repeat, and 24% did not have an explanation documented. The only independent predictor associated with diagnostic paracentesis was being admitted for an acute exacerbation of ascites (OR 3.8, 95% confidence interval (CI) 1.8–7.9) (Table S2). On CPH model, being admitted for any exacerbation of underlying liver disease predicted a longer time to paracentesis (hazard ratio (HR) 2.1, *p* = 0.002), and there were no other independent predictors.

There was no significant difference in median LOS (5 days vs. 5 days, *p* = 0.8) or in‐hospital mortality (2% vs. 4%, *p* = 0.2) between groups. Given the low overall number of mortality events (3.5%) compared to the size of the cohort, we did not attempt to assess predictors of mortality.

### 3.4 Secondary Outcomes

There were significantly less all‐cause 30‐day ED encounters in the order set group (32% vs. 46%, *p* = 0.006) despite this patient cohort having a higher prevalence of decompensated cirrhosis, higher MELD scores and increased cirrhosis‐related complications at baseline (ascites, SBP, and HE). We found similar 30‐day readmission rates between groups (30% vs. 39%, *p* = 0.1) (Table 3). Use of the order set and a history of ascites (whether admitted for an acute exacerbation or not) protected against 30‐day ED encounters on multivariate modelling. For 30‐day all‐cause readmissions, use of the order set and history of ascites were protective against readmission while a history of HE was an independent predictor of readmission (Table S3). There was no significant difference in liver‐specific ED encounters or readmissions between groups at 30 days (Table 3, Table S4). Notably, ED visits and readmissions at outside institutions were not captured due to EMR limitations.

## 4 Discussion

The cirrhosis patient population is particularly vulnerable, with high hospitalization and readmission rates [3, 4]. The increasing prevalence of liver disease and associated healthcare costs highlight an opportunity for improvement in inpatient management of cirrhosis. Standardized order sets have previously been associated with decreased medical errors and mortality [21, 22]. Using pre‐intervention data and evidence‐based clinical practice guidelines, we (1) identified potential areas for improvement to promote adherence to standardized guidelines, and (2) created an admission order set to optimize inpatient practice patterns. Our post‐intervention data revealed that use of the order set was associated with higher adherence to a low sodium, high protein diet and more timely performance of diagnostic paracentesis. Finally, order set use was protective against 30‐day all‐cause ED visits, though it had a less significant impact on additional outcome measures including readmissions.

Strengths of our project include its retrospective cohort design, cost‐effectiveness, high utilization of the order set, and easy replicability at other institutions. Additionally, our findings support previous literature that links standardized order sets and EMR templates to improved evidence‐based care. Mayorga and Rockey looked at patients with known or suspected cirrhosis presenting to the ED with concern for an upper GIB. They found that overall use of antibiotics, time to antibiotics and time to octreotide were all shortened with use of the order set, but found no difference in standardized hospital outcome metrics (i.e., LOS, readmissions, etc.) between groups [23]. Sherman et al. performed a prospective cohort study comparing outcomes among patients with cirrhosis with and without use of a standardized EMR note template. Implementation of an EMR note template with cirrhosis best practices was associated with lower 30‐day mortality and higher rates of diagnostic paracentesis; however, it did not impact readmissions, in‐hospital mortality, 90‐day mortality or LOS [24]. Notably, the sample size was small at 108 patients (83 intervention and 25 control patients). Additionally, baseline characteristics apart from MELD score and other elements of the EMR template (diuretics, SBP prophylaxis, etc.) weren't detailed, making it difficult to interpret potential confounders and external validity of the study.

Bhavsar‐Burke et al. compared pre‐ and post‐intervention data after implementation of a cirrhosis order set resulting in increased utilization of diagnostic paracentesis, low sodium diet, and social work involvement. There were also decreases in LOS and in‐hospital infection, but there was no effect on 30‐ or 90‐day readmissions. In contrast to our study, this was a pre‐post intervention design utilizing a historical control group, and groups were not compared over the same time period [8]. This likely made it more difficult to account for outside confounders or changes that occurred during the intervention period, potentially obscuring the true effect. Bhavsar‐Burke et al.'s historical control group compared to our direct control group highlights the unique design and unprecedented nature of our study. Additionally, Bhavsar‐Burke et al.'s intervention incorporated a ‘pop‐up’ or interruptive alert system that prompted providers to recognize patients admitted with cirrhosis and ascites. Potential downsides to this strategy include false‐negative alerts and alert fatigue. Our stand‐alone order set coupled with provider education is a simpler, more easily reproducible intervention that can quickly be adopted at other institutions and applied to a wide array of diagnoses.

The aforementioned studies show that EMR interventions are useful in improving inpatient, guideline‐directed care; however, the impact on readmission rates and long‐term outcome measures may be limited. Similarly, our data showed that use of a cirrhosis order set had a significant impact on inpatient management and adherence to guideline‐directed care. Even when correcting for other variables, order set use was independently predictive of low sodium and high protein diet orders, underscoring the impact of order set use on clinical decision making. This is important because both low sodium and high protein diets have been linked to improved cirrhosis patient outcomes and potentially mortality [16, 25, 26]. While reducing sodium and increasing protein intake can help manage fluid retention, dietary choices are also key to reducing malnutrition and sarcopenia in the cirrhosis population [13]. By providing hands‐on education on nutrition and dietary choices to patients and their families during hospitalization, this may lessen the burden of education in the outpatient setting.

Time to diagnostic paracentesis in patients with cirrhosis and ascites has consistently been associated with reduced LOS and lower risk of in‐hospital mortality [9, 14, 27, 28, 29]. Our post‐intervention data showed that among patients with a history of ascites, those in the order set group had significantly shorter time to paracentesis. These results further support that standardizing the admission process promotes adherence to quality metrics. By setting electronic reminders and linking current guidelines, providers may be more likely to prioritize timely diagnostic paracenteses.

While order set use seems to have a heavier impact on medical decision making and inpatient management of cirrhosis, our results show a weaker association between order set use and standardized hospital outcome metrics. It is important to note that the baseline characteristics between groups were not identical; specifically, the order set group had a significantly higher MELD score and more patients with decompensated disease and cirrhosis‐related complications. Interestingly, despite the order set patients being the ‘sicker’ cohort, LOS was similar between groups and in‐hospital mortality was slightly less (though not significant) in the order set group. Similarly, 30‐day readmission rates were overall lower in the order set group. This suggests that when controlling for severity of disease, the order set may have a significant impact on these outcomes. Although not statistically significant, these trends are consistently in favor of the order set cohort despite differences in baseline liver function.

Though there was no significant impact on readmissions, we did see a significant difference in all‐cause 30‐day ED visits between groups. These findings held true on multivariate modelling, which showed that order set use was protective against 30‐day ED visits. While the order set focused on inpatient management of cirrhosis, it also emphasized patient education during admission and at time of discharge. We achieved this through pre‐selecting ‘include cirrhosis education in discharge instructions.’ Real‐time education in the hospital coupled with more formal education at time of discharge may have played a role in decreased ED visits. These findings highlight the need for a more pointed, discharge‐directed educational intervention beyond use of a simple admission order set.

The primary cost of this QI initiative was increased staff time associated with developing and using the order set; however, this investment was offset by improved patient care through more consistent, timely and evidence‐based clinical management. Limitations of our study include its single‐center, non‐randomized design and modest sample size. Since ED visits and readmissions were only captured at a single institution, patterns of post‐discharge care could differ between exposure groups and may have led to differential misclassification. Additionally, baseline characteristics were not perfectly comparable between groups and may be indicative of selection bias, though they were controlled for on multivariate analysis. Despite these limitations, we believe our findings are generalizable to other hospital systems for patients admitted with cirrhosis.

Our findings are supportive of prior QI literature, which shows that order sets can help streamline providers' practice patterns and impact guideline‐directed care. With ongoing education, this QI intervention is not only sustainable, but easily adaptable with changing guidelines. Given its relatively low effort and high impact factor, it could easily be applied to other contexts such as cirrhosis outpatient care or ED visits. We acknowledge that improving LOS and readmission rates remain the gold standard for QI initiatives. Therefore, future work should focus on further standardization of the discharge pathway to help optimize patient education, ensure close outpatient follow‐up and ideally reduce cirrhosis‐related hospitalizations.

## Author Contributions

All authors contributed to the study conception and design. Material preparation and data collection were performed by Laura E. Lavette, Hannah Laird, Ashley Chipoletti, and Mira Sridharan. Data analysis and manuscript drafting/revisions were completed by Laura E. Lavette and Zachary Henry. All authors commented on previous versions of the manuscript and read/approved the final version of the manuscript.

## Funding

The authors have nothing to report.

## Ethics Statement

This study has received ethical oversight and approval from a relevant ethics committee or institutional review board.

## Consent

The authors have nothing to report.

## Conflicts of Interest

The authors declare no conflicts of interest.

## Supporting information


**Table S1:** Univariate and multivariate analyses for low sodium and high protein diets ordered during admission.
**Table S2:** Univariate and multivariate analyses for diagnostic paracenteses performed in patients with a history of ascites.
**Table S3:** Univariate and multivariate analyses for 30‐day ED visits and 30‐day readmissions.
**Table S4:** Univariate and multivariate analyses for Liver‐Related 30‐day ED visits and 30‐day readmissions.

## Data Availability

The data that support the findings of this study are available from the corresponding author upon reasonable request.

## References

[1] H. Devarbhavi , S. K. Asrani , J. P. Arab , Y. A. Nartey , E. Pose , and P. S. Kamath , “Global Burden of Liver Disease: 2023 Update,” Journal of Hepatology 79, no. 2 (2023): 516–537, 10.1016/j.jhep.2023.03.017. 36990226

[2] D. P. Ladner , M. Gmeiner , B. J. Hasjim , et al., “Increasing Prevalence of Cirrhosis Among Insured Adults in the United States, 2012–2018,” PLoS One 19, no. 2 (2024): e0298887, 10.1371/journal.pone.0298887. 38408083 PMC10896513

[3] F. Kanwal , R. Nelson , Y. Liu , et al., “Cost of Care for Patients With Cirrhosis,” American Journal of Gastroenterology 119, no. 3 (2024): 497–504, 10.14309/ajg.0000000000002472. 37561079

[4] A. P. Desai , P. Mohan , B. Nokes , et al., “Increasing Economic Burden in Hospitalized Patients With Cirrhosis: Analysis of a National Database,” Clinical and Translational Gastroenterology 10, no. 7 (2019): e00062, 10.14309/ctg.0000000000000062. 31343469 PMC6708673

[5] E. S. Orman , M. Ghabril , T. W. Emmett , and N. Chalasani , “Hospital Readmissions in Patients With Cirrhosis: A Systematic Review,” Journal of Hospital Medicine 13, no. 7 (2018): 490–495, 10.12788/jhm.2967. 29694458 PMC6202277

[6] E. B. Tapper and M. Volk , “Strategies to Reduce 30‐Day Readmissions in Patients With Cirrhosis,” Current Gastroenterology Reports 19, no. 1 (2017): 1, 10.1007/s11894-017-0543-3. 28101791

[7] F. Kanwal , J. Kramer , S. M. Asch , et al., “An Explicit Quality Indicator Set for Measurement of Quality of Care in Patients With Cirrhosis,” Clinical Gastroenterology and Hepatology 8, no. 8 (2010): 709–717, 10.1016/j.cgh.2010.03.028. 20385251

[8] I. Bhavsar‐Burke , J. J. Guardiola , N. Hamade , et al., “Use of a Cirrhosis Admission Order Set Improves Adherence to Quality Metrics and May Decrease Hospital Length of Stay,” American Journal of Gastroenterology 118, no. 1 (2023): 114–120, 10.14309/ajg.0000000000001930. 35971218

[9] R. Rosenblatt , Z. Tafesh , N. Shen , et al., “Early Paracentesis in High‐Risk Hospitalized Patients: Time for a New Quality Indicator,” American Journal of Gastroenterology 114, no. 12 (2019): 1863–1869, 10.14309/ajg.0000000000000443. 31688022

[10] D. Kumral , R. Qayyum , S. Roseff , R. K. Sterling , and M. S. Siddiqui , “Adherence to Recommended Inpatient Hepatic Encephalopathy Workup,” Journal of Hospital Medicine 14, no. 3 (2019): 157–160, 10.12788/jhm.3152. 30811321

[11] J. S. Bajaj , J. G. O'Leary , P. Tandon , et al., “Targets to Improve Quality of Care for Patients With Hepatic Encephalopathy: Data From a Multi‐Center Cohort,” Alimentary Pharmacology & Therapeutics 49, no. 12 (2019): 1518–1527, 10.1111/apt.15265. 31032966 PMC6538445

[12] M. J. Thomson , A. S. F. Lok , and E. B. Tapper , “Appropriate and Potentially Inappropriate Medication Use in Decompensated Cirrhosis,” Hepatology 73, no. 6 (2021): 2429–2440, 10.1002/hep.31548. 32911564 PMC7943648

[13] J. C. Lai , P. Tandon , W. Bernal , et al., “Malnutrition, Frailty, and Sarcopenia in Patients With Cirrhosis: 2021 Practice Guidance by the American Association for the Study of Liver Diseases,” Hepatology 74, no. 3 (2021): 1611–1644, 10.1002/hep.32049. 34233031 PMC9134787

[14] J. Badal , B. Badal , M. Nawras , et al., “Diagnostic Paracentesis Within 1 Day Is Associated With Reduced Mortality and Length of Hospital Stay in Patients With Cirrhosis and Ascites,” Digestive Diseases and Sciences 69, no. 4 (2024): 1454–1466, 10.1007/s10620-023-08249-w. 38217676

[15] G. Daftari , A. N. Tehrani , F. Pashayee‐khamene , et al., “Dietary Protein Intake and Mortality Among Survivors of Liver Cirrhosis: A Prospective Cohort Study,” BMC Gastroenterology 23, no. 1 (2023): 227, 10.1186/s12876-023-02832-1. 37400778 PMC10316618

[16] F. Pashayee‐Khamene , M. Hajimohammadebrahim‐Ketabforoush , M. Saber‐Firoozi , et al., “Salt Consumption and Mortality Risk in Cirrhotic Patients: Results From a Cohort Study,” Journal of Nutritional Science 11 (2022): e99, 10.1017/jns.2022.69. 36405096 PMC9672831

[17] S. W. Biggins , P. Angeli , G. Garcia‐Tsao , et al., “Diagnosis, Evaluation, and Management of Ascites, Spontaneous Bacterial Peritonitis and Hepatorenal Syndrome: 2021 Practice Guidance by the American Association for the Study of Liver Diseases,” Hepatology 74, no. 2 (2021): 1014–1048, 10.1002/hep.31884. 33942342

[18] N. Patel , S. Silvey , J. G. O'Leary , et al., “Early Paracentesis Is Associated With Better Prognosis Compared With Late or No‐Paracentesis in Hospitalized Veterans With Cirrhosis and Ascites,” Liver Transplantation 29, no. 9 (2023): 919–927, 10.1097/LVT.0000000000000137. 36971257 PMC10523869

[19] G. Ogrinc , L. Davies , D. Goodman , P. Batalden , F. Davidoff , and D. Stevens , “SQUIRE 2.0 (Standards for QUality Improvement Reporting Excellence): Revised Publication Guidelines From a Detailed Consensus Process,” BMJ Quality and Safety 25, no. 12 (2016): 986–992, 10.1136/bmjqs-2015-004411. PMC5256233 26369893

[20] S. Cuschieri , “The STROBE Guidelines,” Saudi Journal of Anaesthesia 13, no. Suppl 1 (2019): S31–S34, 10.4103/sja.SJA_543_18. 30930717 PMC6398292

[21] S. R. Pendharkar , M. B. Ospina , D. A. Southern , et al., “Effectiveness of a Standardized Electronic Admission Order Set for Acute Exacerbation of Chronic Obstructive Pulmonary Disease,” BMC Pulmonary Medicine 18, no. 1 (2018): 93, 10.1186/s12890-018-0657-x. 29843772 PMC5975274

[22] C. Wells and H. Loshak , Standardized Hospital Order Sets in Acute Care: A Review of Clinical Evidence, Cost‐Effectiveness, and Guidelines (Canadian Agency for Drugs and Technologies in Health, 2019), accessed June 4, 2024, http://www.ncbi.nlm.nih.gov/books/NBK546326/. 31525009

[23] C. A. Mayorga and D. C. Rockey , “Clinical Utility of a Standardized Electronic Order Set for the Management of Acute Upper Gastrointestinal Hemorrhage in Patients With Cirrhosis,” Clinical Gastroenterology and Hepatology 11, no. 10 (2013): 1342–1348, 10.1016/j.cgh.2013.04.021. 23639605

[24] Z. Sherman , N. Wahid , M. Wagner , et al., “Integration of Cirrhosis Best Practices Into Electronic Medical Record Documentation Associated With Reduction in 30‐Day Mortality Following Hospitalization,” Journal of Clinical Gastroenterology 57, no. 9 (2023): 951–955, 10.1097/MCG.0000000000001787. 36730665

[25] G. R. Swart , J. W. O. van den Berg , J. K. van Vuure , T. Rietveld , D. L. Wattimena , and M. Frenkel , “Minimum Protein Requirements in Liver Cirrhosis Determined by Nitrogen Balance Measurements at Three Levels of Protein Intake,” Clinical Nutrition 8, no. 6 (1989): 329–336, 10.1016/0261-5614(89)90008-3. 16837309

[26] G. R. Swart , J. W. van den Berg , J. L. Wattimena , T. Rietveld , J. K. van Vuure , and M. Frenkel , “Elevated Protein Requirements in Cirrhosis of the Liver Investigated by Whole Body Protein Turnover Studies,” Clinical Science (London, England) 75, no. 1 (1988): 101–107, 10.1042/cs0750101. 3409620

[27] A. Beran , M. F. H. Mohamed , A. Vargas , et al., “Early Diagnostic Paracentesis Improves Outcomes of Hospitalized Patients With Cirrhosis and Ascites: A Systematic Review and Meta‐Analysis,” Official Journal of the American College of Gastroenterology | ACG 119 (2024): 2259–2266, 10.14309/ajg.0000000000002906. 38916217

[28] J. J. Kim , M. M. Tsukamoto , A. K. Mathur , et al., “Delayed Paracentesis Is Associated With Increased In‐Hospital Mortality in Patients With Spontaneous Bacterial Peritonitis,” American Journal of Gastroenterology 109, no. 9 (2014): 1436–1442, 10.1038/ajg.2014.212. 25091061

[29] E. S. Orman , P. H. Hayashi , R. Bataller , and A. S. Barritt , “Paracentesis Is Associated With Reduced Mortality in Patients Hospitalized With Cirrhosis and Ascites,” Clinical Gastroenterology and Hepatology 12, no. 3 (2014): 496–503.e1, 10.1016/j.cgh.2013.08.025. 23978348 PMC3944409

