# Supplementary material for: Standardization of the Cirrhosis Admission Process and Its Impact on Inpatient Management and Patient Outcomes
Source: Liver Int. 2026 May 5;46:e70674. doi: 10.1111/liv.70674 (PMC13140773; doi:10.1111/liv.70674)
Supplement: Supplementary file 1 — Table S1: Univariate and multivariate analyses for low sodium and high protein diets ordered during admission. Table S2: Univariate and multivariate analyses for diagnostic paracenteses performed in patients with a history of ascites. Table S3: Univariate and multivariate analyses for 30‐day ED visits and 30‐day readmissions. Table S4: Univariate and multivariate analyses for Liver‐Related 30‐day ED visits and 30‐day readmissions. [file LIV-46-0-s001.docx]

**SUPPLEMENTAL TABLES**

**Table S1.** Univariate and multivariate analyses for low sodium and high protein diets ordered during admission.

|  | **Univariate** | | **Multivariate (OR [95% CI])** |
| --- | --- | --- | --- |
| **Low Sodium Diet Ordered** | **OR (95% CI)** | **p-value** |  |
| MELD-3.0 | 1.04 (1.02 – 1.06) | 0.001 | --- |
| Order Set Use | 22.2 (11.9 – 41.5) | 0.001 | **23.6 [12.1-45.9]** |
| Decompensated Cirrhosis | 4.3 (2.1 – 8.7) | 0.001 | --- |
| History of HE | 2.6 (1.7 – 3.9) | 0.001 | --- |
| History of SBP | 2.4 (1.2 – 4.8) | 0.01 | --- |
| History of Ascites | 5.3 (3.4 – 8.3) | 0.001 | **6.0 [3.5-10.3]** |
| Admitted for Ascites | 5.0 (2.3 – 10.8) | 0.001 | --- |
| Admitted for Liver Disease | 2.6 (1.7 – 3.9) | 0.001 | --- |
|  | | | |
| **High Protein Diet Ordered** | **OR (95% CI)** | **p-value** |  |
| MELD-3.0 | 1.05 (1.03 – 1.07) | 0.001 | **1.04 [1.02-1.1]** |
| Order Set Use | 16.3 (9.2 – 28.7) | 0.001 | **15.2 [8.4-27.4]** |
| Decompensated Cirrhosis | 4.7 (2.3 – 9.8) | 0.001 | --- |
| History of HE | 2.3 (1.5 – 3.5) | 0.001 | --- |
| History of Ascites | 3.2 (2.1 – 4.9) | 0.001 | **2.2 [1.3-3.7]** |
| Admitted for Ascites | 3.0 (1.6 – 5.9) | 0.001 | --- |
| Admitted for Liver Disease | 2.1 (1.4 – 3.2) | 0.001 | --- |

**Table S2.** Univariate and multivariate analyses for diagnostic paracenteses performed in patients with a history of ascites.

|  | **Univariate (p-value)** | | **Multivariate (OR [95% CI])** |
| --- | --- | --- | --- |
| **Diagnostic Paracentesis** | **OR (95% CI)** | **p-value** |  |
| Admitted for Ascites | 3.7 (1.8 – 7.9) | 0.001 | **3.8 [1.8-7.9]** |
| Admitted for Liver Disease | 2.4 (1.3 – 4.4) | 0.009 | --- |
| INR | 0.79 (0.59 – 1.04) | 0.09 | --- |

**Table S3.** Univariate and multivariate analyses for 30-day ED visits and 30-day readmissions.

|  | **Univariate (p-value)** | | **Multivariate (OR [95% CI])** |
| --- | --- | --- | --- |
| **30-Day ED Encounter** | **OR (95%CI)** | **p-value** |  |
| Order Set Use | 0.55 (0.36 – 0.84) | 0.006 | **0.62 [0.40-0.95]** |
| Low Sodium Diet | 0.64 (0.42 – 0.97) | 0.04 | --- |
| History of Ascites | 0.48 (0.32 – 0.73) | 0.001 | **0.52 [0.34-0.79]** |
| Admitted for Ascites | 0.54 (0.29 – 0.99) | 0.05 | --- |
| Admitted for Liver Disease | 0.69 (0.46 – 1.04) | 0.08 | --- |
|  | | | |
| **30-Day Readmissions** | **OR (95%CI)** | **p-value** |  |
| Order Set Use | 0.67 (0.44 – 1.02) | 0.06 | **0.62 [0.40 – 0.98]** |
| Low Sodium Diet | 0.64 (0.42 – 0.99) | 0.04 | --- |
| History of HE | 1.5 (0.98 – 2.35) | 0.06 | **1.8 [1.2 – 2.9]** |
| History of Ascites | 0.63 (0.41 – 0.96) | 0.03 | **0.63 [0.41 – 0.97]** |
| Admitted for Ascites | 0.49 (0.25 – 0.94) | 0.03 | --- |
| Admitted for HE | 1.6 (0.95 – 2.7) | 0.07 | --- |
| Age | 1.02 (1.00 – 1.03) | 0.07 | --- |

**Table S4.** Univariate and multivariate analyses for Liver-Related 30-day ED visits and 30-day readmissions.

|  | **Univariate (p-value)** | | **Multivariate (OR [95% CI])** |
| --- | --- | --- | --- |
| **30-Day Liver-Related ED Encounter** | **OR (95%CI)** | **p-value** |  |
| History of HE | 1.86 [1.07 - 3.23] | 0.03 | --- |
| Admitted for HE | 2.44 [1.35-4.39] | 0.003 | **2.44 [1.35-4.39]** |
| Admitted for Liver Disease | 2.05 [1.19 - 3.55] | 0.01 | --- |
|  | | | |
| **30-Day Liver-Related Readmissions** | **OR (95%CI)** | **p-value** |  |
| Decompensated State | 4.31 [1.01 - 18.37] | 0.05 | --- |
| Admitted for Liver Disease | 2.0 [1.15 - 3.46] | 0.01 | --- |
| History of HE | 2.33 [1.30 - 4.15] | 0.004 | --- |
| Admitted for HE | 3.26 [1.83 - 5.83] | <0.001 | **3.26 [1.83 – 5.83]** |
